# The expression profiles of circular RNAs and competing endogenous RNA networks in intrahepatic cholangiocarcinoma

**DOI:** 10.3389/fcell.2022.942853

**Published:** 2022-10-07

**Authors:** Zi Liang, Liyan Liu, Xinyi Guo, Xia Wu, Yun-Li Yu, Ziyang Yu, Xiaolong Hu, Xing Zhang, Ji Wang

**Affiliations:** ^1^ Department of Oncology, The Second Affiliated Hospital of Soochow University, Suzhou, China; ^2^ School of Biology and Basic Medical Science, Soochow University, Suzhou, China; ^3^ Department of Blood Transfusion, The Affiliated Changzhou No. 2 People’s Hospital of Nanjing Medical University, Changzhou, China; ^4^ Department of Clinical Pharmacology, The Second Affiliated Hospital of Soochow University, Suzhou, China; ^5^ Department of Gynecology and Obstetrics, The Second Affiliated Hospital of Soochow University, Suzhou, China

**Keywords:** intrahepatic cholangiocarcinoma, circular RNAs, competing endogenous RNA, expression pattern, regulatory networks

## Abstract

**Introduction:** Intrahepatic cholangiocarcinoma (iCCA) is a heterogeneous entity with diverse etiologies, morphologies, and clinical outcomes, but our knowledge of its epidemiology and carcinogenesis is very limited.

**Materials and methods:** The expression patterns of circRNAs were explored in iCCA tissues and corresponding adjacent normal ones, denoted by (iCCA) and (iCCAP), respectively, using high-throughput sequencing.

**Results:** A total of 117 differential expressed (DE) circRNAs were identified. Based on the parental transcripts of circRNAs, these DE circRNAs were related to several important GO terms and were enriched in important pathways. Two circRNA-mediated ceRNA networks were constructed and many important metabolic pathways related to mRNAs were regulated by DE circRNAs *via* miRNAs.

**Conclusion:** Our study revealed the DE circRNAs in the iCCA tissues compared with iCCAP ones, suggesting that circRNAs may play crucial roles in the pathogenesis of iCCA.

## Introduction

Cholangiocarcinoma is classified into three subtypes based on the site of the anatomic origin: intrahepatic cholangiocarcinoma (iCCA), perihilar cholangiocarcinoma (pCCA), and distal cholangiocarcinoma (dCCA) (Wu et al., 2019b). Following hepatocellular carcinoma (HCC), iCCA is regarded as the second most common malignancy that occurs in the liver with an increasing incidence and high-case mortality worldwide (Shaib et al., 2004; Are et al., 2017; Krasinskas, 2018). Unlike the HCC, most iCCA develop in a non-cirrhotic liver with no or nonspecific symptoms (Krasinskas, 2018), which makes the diagnosis of iCCA in its early stage a huge challenge. Most of the patients are diagnosed at an advanced stage and only approximately 20% of the cases are surgically resectable (Chun and Javle, 2017). Therefore, it is of great importance and necessity to search for novel biomarkers and explore the molecular mechanism of oncogenesis in iCCA.

Circular RNAs (circRNAs) represent a kind of novel RNA transcripts, which are generated by back-splicing events. Since they have a circular structure, it is not easy to digest them by the endonuclease. An increasing amount of data shows that circRNAs are important regulatory molecules that play crucial roles in the development and progression of many diseases, including HCC, cancers, atherosclerosis, and carcinomas. The biological function of circRNAs mainly includes the miRNA sponge, RNA-binding proteins, and translation (Kristensen et al., 2019). A piece of emerging evidence has recently demonstrated that circRNAs are not only related to the development and progression of diseases (Li et al., 2019a; Bai et al., 2019; Li et al., 2019b; Zhou et al., 2019), but they also act as biomarkers for the diagnosis of diseases (Wang et al., 2019a; Wu et al., 2019a; Shao et al., 2019; Tian et al., 2019). Most of the studies showed that circRNAs identified from iCCA served as endogenous RNA for miRNAs sponge. The expression level of circ_0059961 was reduced in iCCA tissues. Overexpression of circ_0059961 decreased the tumor cell proliferation, migration, and invasion by modulating the miR-629-5p/SFRP2 axis (Zhang et al., 2022) (). The expression level of circSETD3 (hsa_circ_0000567) was decreased in iCCA tissues and cell lines. Overexpression of hsa_circ_0000567 inhibited proliferation and induced apoptosis in iCCA cells by mediating the miR-421/B-cell lymphoma-2 modifying factor axis (Xiong et al., 2022). The expression level of circ_0000591 was upregulated in iCCA cells and tissues, which served as endogenous RNA for miR-326 to the progression of iCCA *via* miR-326/TLR4/MyD88/IL6 axis (Xiao et al., 2022). Circ_0020256 promoted the proliferation, migration, and invasion of iCCA cells *via* a Circ_0020256/miR-432-5p/E2F3 axis (Chen et al., 2022). In addition, a novel protein cGGNBP2-184aa was translated from circRNA GGNBP2 in iCCA, which promoted iCCA cell proliferation and metastasis *via* modulating IL-6/STAT3 signaling (Li et al., 2022). At present, the pathogenesis of iCCA is still vague.

In this experiment, the expression patterns of circRNAs in iCCA tissues along with adjacent normal tissues, denoted by (iCCA) and (iCCAP), respectively, were identified using transcriptome sequencing, and the regulatory mechanism of circRNAs was explored in iCCA. A total of 16084 circRNAs were identified from the samples of iCCA and iCCAP, among which 151 differential expressed (DE) circRNAs were identified, which included 121 upregulated and 30 downregulated circRNAs. The competitive endogenous RNA (ceRNA) networks indicated that many important metabolic pathways that are related to mRNAs were regulated by DE circRNAs *via* miRNA in the progress or pathogenesis of iCCA. These findings will provide new insights into the regulatory roles of circRNAs in the epidemiology and carcinogenesis of iCCA.

## Materials and methods

### Patients and tissue samples preparation

**FIGURE 1:**
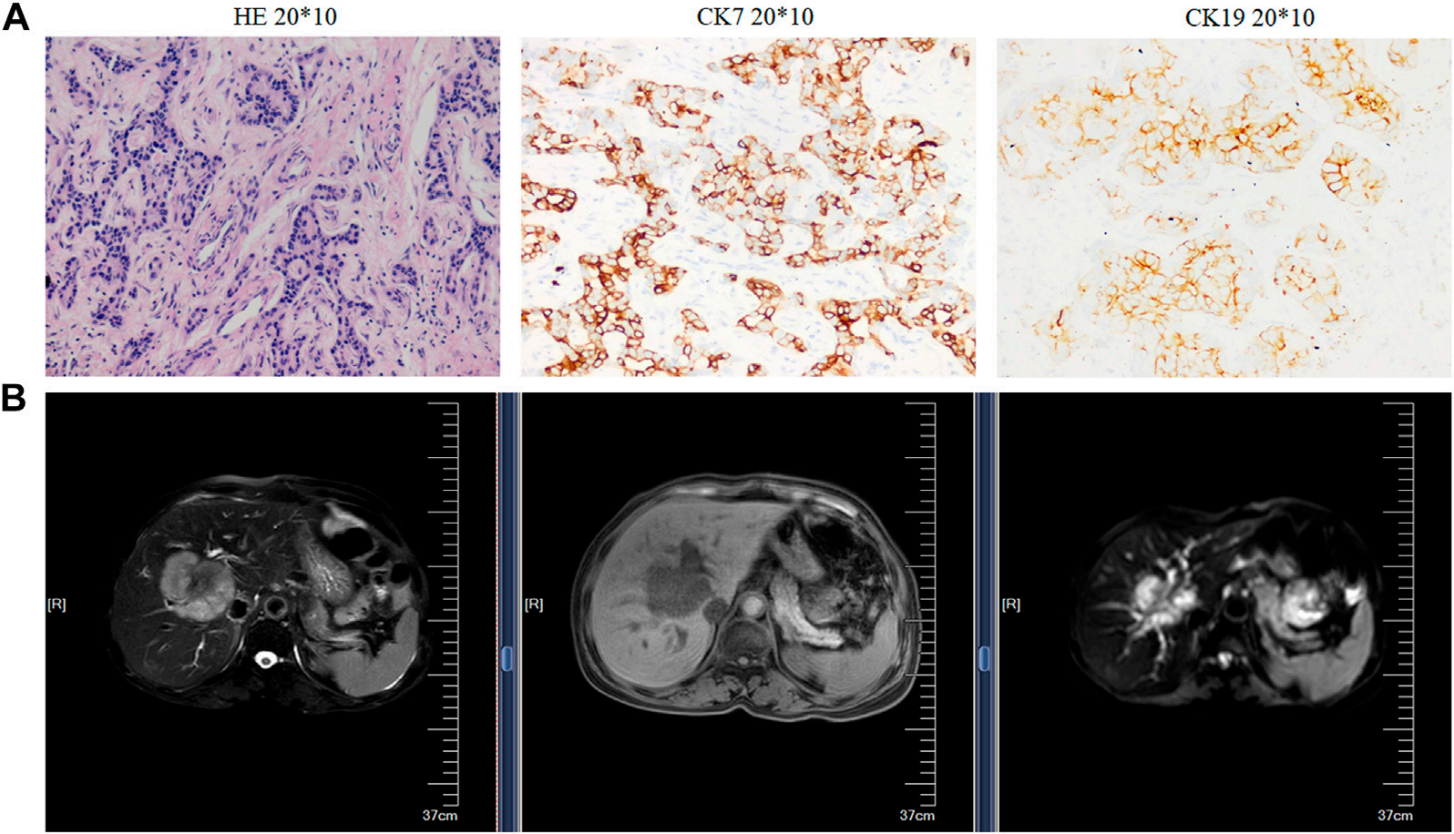
Histopathological and radiographic analysis. **(A)** Hematoxylin and eosin (HE) staining (left), the immunohistochemical images, and expression rate of CK7 (middle) and CK19 (right). **(B)** Radiographic.

The resected iCCA and iCCAP tissues, all confirmed by at least two pathologists, were collected from 10 iCCA patients who were diagnosed at the Second Affiliated Hospital of Soochow University from May 2018 to April 2019. Before the surgical resection, none of the iCCA patients had received adjuvant treatment. All the iCCA and iCCAP samples were resected from the iCCA patients and quickly frozen in liquid nitrogen. The samplings of the iCCA tissues (iCCA1, iCCA2, and iCCA3) and corresponding adjacent normal tissues (iCCAP1, iCCAP2, and iCCAP3) had three biological replicates, each made up of the tissues of three different patients. The pieces of the cancer tissues from each patient were fixed in 4% paraformaldehyde and sectioned at 5 μm for hematoxylin and eosin (HE) staining. The proliferation index and tumor differentiation were verified (Figure 1A), and the radiographic finding was also used to verify the iCCA (Figure 1B). This experiment was approved by the Ethics Committee of the Second Affiliated Hospital of Soochow University, and written informed consent was obtained from all the subjects.

### RNA sequencing and data preprocessing

Three iCCA and iCCAP samples were chosen to perform the RNA sequencing analysis. The TRIzol reagent was used to extract the total RNAs from the collected tissues following the manufacturer’s protocol. After the quality and quantity control, the RNA integrity number of each sample (RIN) ≥ 9 was subjected to the RNA sequencing analysis. The TruSeq Stranded Total RNA with the Ribo-Zero Gold kit (Ambion, Foster City, CA, United States) was used to construct the libraries. Six libraries were conducted on the HiSeqTM 2500 sequencing platform (Illumina) (by Shanghai OE Biotech, Shanghai, China). The raw reads from the sequencing were filtered based on the quality to get high-quality clean reads using the Trimmomatic software (Bolger et al., 2014). The clean reads from the quality control were aligned with the human reference genome (GRCh38.p12) using the hisat2 software (Kim et al., 2015). Finally, the raw data from the whole transcriptome sequencing and small RNA sequencing were submitted to the NCBI Sequence Read Archive with accession numbers PRJNA763017 and PRJNA763019.

### CircRNA prediction and bioinformatics analysis

Based on the junction reads and the GT-AG cleavage signals, the complete sequences of the circRNAs were predicted using the CIRI software (Gao et al., 2015). The expression levels of the circRNAs were quantified using the RPM (spliced reads per million) algorithm while normalizing the number of junction reads and fold change by DESeq (http://bioconductor.org/packages/release/bioc/html/DESeq.html) with the screen parameters (*p*-values ≤ 0.05 and fold change ≥ 2). The Gene Ontology (GO) annotation and Kyoto Encyclopedia of Genes and Genomes (KEGG) enrichment of the DE circRNAs were analyzed with the parental transcripts of these circRNAs using the Hypergeometric Distribution Test.

### miRNA prediction and circRNA-miRNA interaction analysis

CircRNAs can serve as miRNA target molecules, and the circRNA/miRNA interactions were predicted using the miRanda software (John et al., 2004). To investigate the regulatory mechanism of circRNA-miRNA, their co-expression network was constructed based on the *p*-value of the hypergeometric distribution, which was calculated and ranked using miRanda between the circRNA and miRNA (Zhang et al., 2017). The top 300 co-expressions (lowest *p*-values) were chosen to construct the circRNA-miRNA interaction network using the Cytoscape software (version 3.7.1).

### ceRNA network analysis

The competitive endogenous RNA (ceRNA) hypothesis is a novel approach to uncover the molecular pathogenesis of diseases (Salmena et al., 2011). In order to identify the potential interaction relationship among DE RNAs (circRNAs, miRNAs, and mRNAs) in iCCA, two ceRNA networks were constructed using the DE circRNAs and mRNAs from the whole transcription sequencing and the DE miRNAs from the small RNA sequencing. The parameters for the correlation between the miRNAs and target mRNAs were correlation ≥ 0.7 & *p*-value ≤ 0.05, and the ceRNAs were constructed based on the *p*-value ≤ 0.05. The DE circRNA-mediated ceRNAs were further analyzed through the GO and KEGG analysis.

### Real-time PCR assay

To verify the authenticity of the DE circRNAs that were identified from the iCCA, 7 DE circRNAs (hsa_circ_0006114, hsa_circ_0015839, hsa_circ_0058010, hsa_circ_0000020, hsa_circ_0001727, hsa_circ_0006633, and hsa_circ_0026920) were randomly selected for validation with the real-time PCR. According to the flanking sequences of the junction sites of these circRNAs, the divergent primers were designed to do the validation (Table 1). After extracting the total RNAs from the iCCA and iCCAP samples, as mentioned above, cDNA was obtained using the EasyScript cDNA Synthesis SuperMix (Transgen Biotech, Beijing, China). The expression levels of these DE circRNAs were detected using the real-time PCR in iCCA (*n* = 10) and iCCAP (*n* = 10). The real-time PCR was done using the Real-Time PCR Detection System (Bio-Rad, United States) with the Universal SYBR Green Supermix (Bio-Rad, United States). We used β-actin (internal standard control) as the reference gene to calculate the expression levels of the circRNAs using the 2^−ΔΔCt^ method (Livak and Schmittgen, 2001). All the experiments were repeated in triplicate.

**TABLE 1:** The primers for real-time PCR.

Primer name	Sequence (5′-3′)
hsa_circ_0006114-F	CTT​ATG​AAC​TTG​AGA​CAC​C
hsa_circ_0006114-R	GAA​CAG​CAC​CTG​GGA​CTG​A
hsa_circ_0015839-F	TCC​AGG​AGG​TCA​AAA​GAA​AAT
hsa_circ_0015839-R	GTG​GTT​TGA​TGT​GTT​CCT​GGT
hsa_circ_0058010-F	GGCATTTACAGGAGGTTT
hsa_circ_0058010-R	CAG​ACT​CCA​TGG​TAC​TTG​GC
hsa_circ_0000020-F	CCA​GAG​GAG​ATT​GCA​GAC​CA
hsa_circ_0000020-R	CCT​CAA​TGT​TCT​GTT​GCC​TGC
hsa_circ_0001727-F	CTC​TTA​CAG​TCA​CGA​GGA​A
hsa_circ_0001727-R	GCT​CAC​CTT​TAT​GTC​CTG​GG
hsa_circ_0006633-F	GAT​TTG​CTG​GGA​TAA​GGC​GG
hsa_circ_0006633-R	TCCCTTGTACAACTTTCG
hsa_circ_0026920-F	CCT​CTT​CCT​GCT​CTT​TGT​GC
hsa_circ_0026920-R	GGG​AGC​CGT​TCA​GAC​ATG​G

### Statistical analysis

GraphPad Prism software version 5.0 (GraphPad Software, San Diego, CA, United States) was used to draw all the figures. The Student-T test was used for the statistical analysis. The following symbols were used: *** for *p* < 0.001, ** for *p* < 0.01, and * for *p* < 0.05.

## Results

### Identification and quantification of circRNAs in iCCA

**FIGURE 2:**
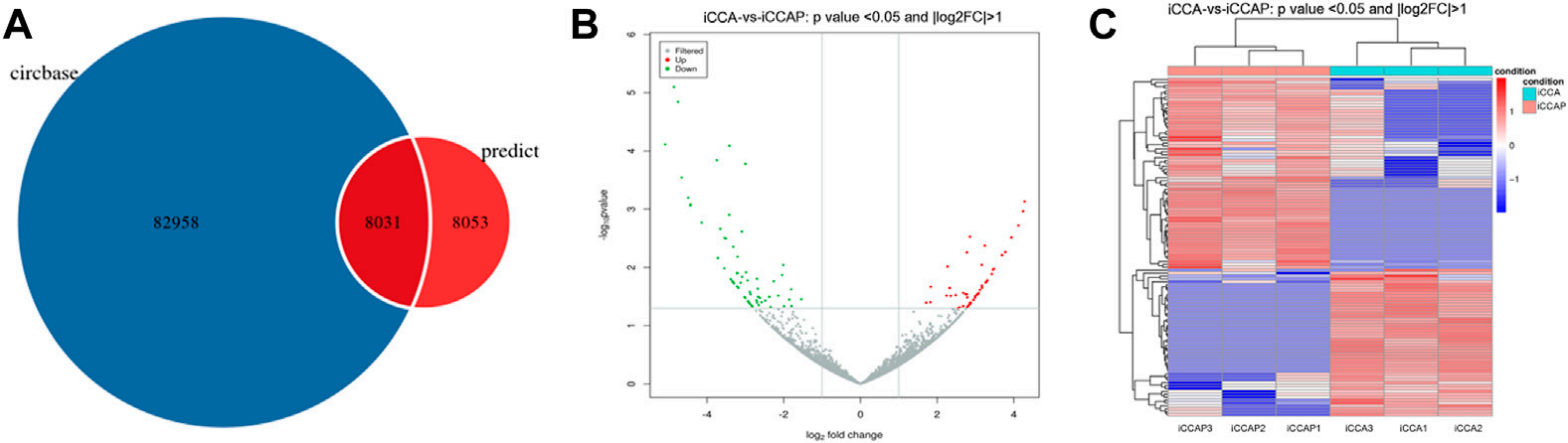
Identification of the DE circRNAs from the iCCA vs. iCCAP. **(A)** Total circRNAs identified from the six samples were compared with the CircBase. **(B)** Volcano plots showing the DE circRNAs in iCCA and iCCAP. The red and green points represent the DE circRNAs. **(C)** Heatmaps representing the mean-centered and normalized data relative to the DE circRNAs in iCCA and iCCAP with *p*-value < 0.05 and log2FC > 1.

To obtain the expression patterns of the circ-RNAs between iCCA and iCCAP, three iCCA and iCCAP samples from the patients were used for the RNA sequencing. These iCCA patients were diagnosed based on clinicopathological information. Compared with the public circRNA datasets in CircBase, a total of 16084 circRNAs were identified from the iCCA and iCCAP, among which 8031 circRNAs had been reported in the CircBase datasets, while 8053 circRNAs were not collected in the public CircBase as the novel circRNAs (Figure 2A). The differential expression of the circRNAs was visualized in the volcano plots (Figure 2B). The clustering analysis of the differential expression of circRNA in the different samples was displayed in a heatmap (Figure 2C). The volcano plots and heatmap revealed the different expression profiles of the circRNAs between iCCA and iCCAP. There were 117 DE circRNAs (Fold Change > 2 and *p*-value < 0.05) in the iCCA tissues compared with the iCCAP ones, including 51 upregulated and 66 downregulated circRNAs. Based on the fold changes and *p*-value, the top 10 DE circRNAs are shown in Table 2.

**TABLE 2:** The top10 DE circRNAs identified from the iCCA vs. iCCAP.

circRNA_id	FoldChange	log2FoldChange	*p*-value	Up/down
circRNA_12475	19.4281641	4.280077672	0.000736	Up
circRNA_06209	18.91693655	4.241606569	0.001084	Up
circRNA_02984	17.36968858	4.118499983	0.001902	Up
circRNA_08944	15.31046183	3.936445897	0.003049	Up
circRNA_01258	13.73555399	3.779843194	0.005419	Up
circRNA_10575	12.9231845	3.691889714	0.006118	Up
circRNA_14918	11.18185365	3.483087463	0.010492	Up
circRNA_08874	10.97411432	3.456032604	0.010843	Up
circRNA_15254	10.75559902	3.427015971	0.012977	Up
circRNA_06892	9.91579575	3.309728554	0.016615	Up
circRNA_01537	0.074583551	−3.744998694	0.000143	Down
circRNA_00329	0.056778262	−4.1385175	0.001695	Down
circRNA_00074	0.046463725	−4.427751355	0.000825	Down
circRNA_10816	0.046255372	−4.434235252	0.000862	Down
circRNA_06019	0.044534032	−4.488947968	0.000637	Down
circRNA_07548	0.039537933	−4.660618756	0.000285	Down
circRNA_03800	0.037030969	−4.755123901	1.43E-05	Down
circRNA_02278	0.034387662	−4.861965173	7.95E-06	Down
circRNA_06028	0.029343409	−5.090819717	7.69E-05	Down
circRNA_03621	0.029006917	−5.107459204	1.58E-06	Down

### Validation of differential expressed circRNAs

**FIGURE 3:**
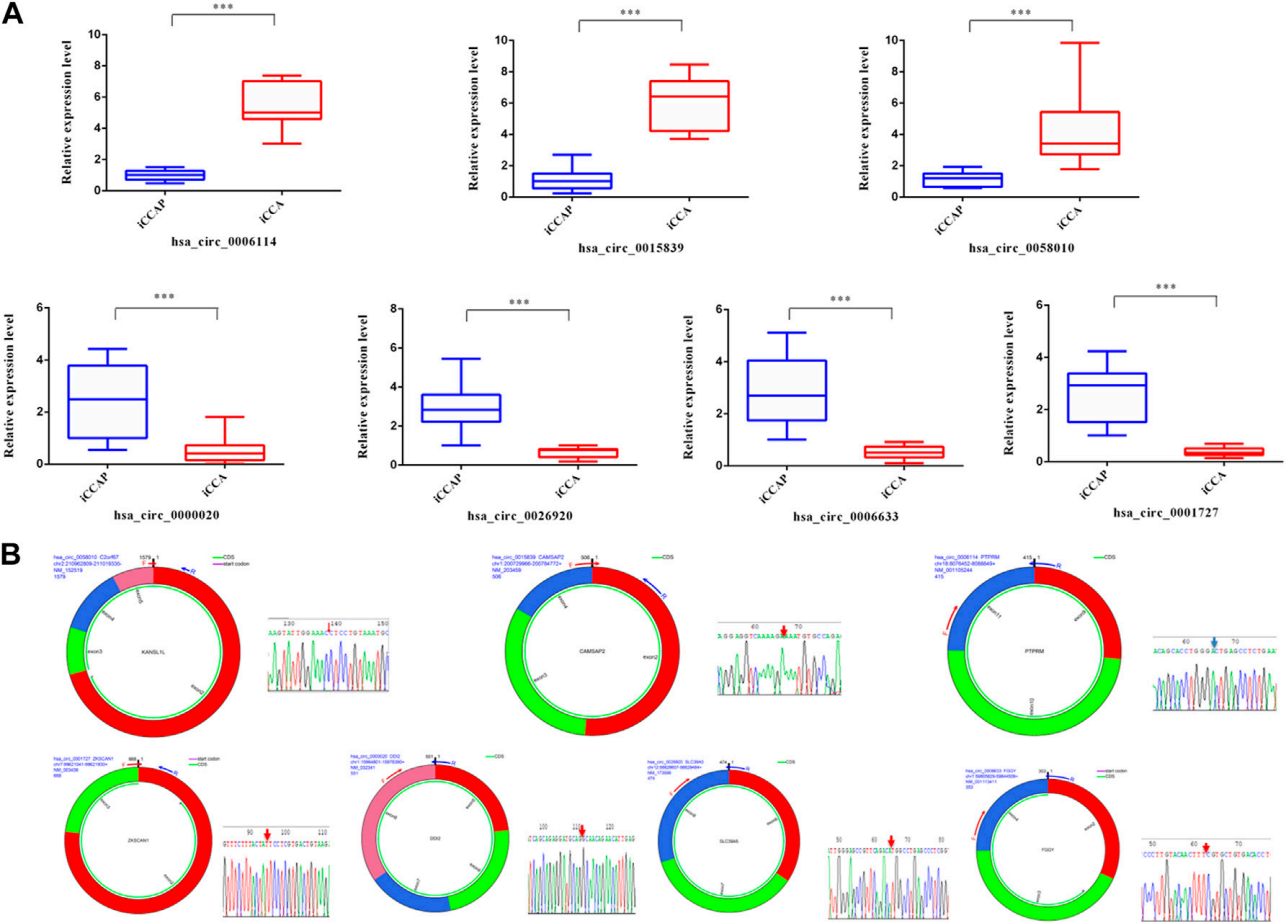
Validation of circRNAs with real-time PCR and sequencing. **(A)** The expression levels of circRNAs (hsa_circ_0006114, hsa_circ_0015839, hsa_circ_0058010, hsa_circ_0000020, hsa_circ_0001727, hsa_circ_0006633, and hsa_circ_0026920) were detected using real-time PCR. **(B)** Sanger sequencing of seven PCR products resulting from divergent primers demonstrating the head-to-tail junction.

To validate the truth of the RNA sequencing data, 7 DE circRNAs were randomly selected from the sequencing data for validation. The results from the real-time PCR were consistent with the RNA sequencing data with a similar tendency (Figure 3A). The junction sites of these circRNAs were also detected using agarose gel electrophoresis and Sanger sequencing (Figure 3B).

### Functional annotation of differential expressed circRNAs

**FIGURE 4:**
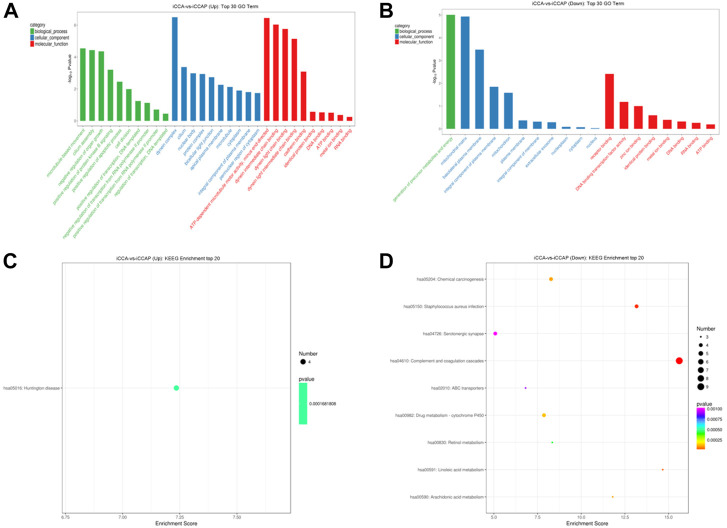
GO annotation and KEGG pathway analysis of the DE circRNAs. **(A)** GO annotation of the upregulated circRNAs with parental transcripts. **(B)** GO annotation of the downregulated circRNAs with parental transcripts. Only the top 30 GO terms are shown in the figures. **(C)** KEGG pathways analysis of the upregulated circRNAs with parental transcripts. **(D)** KEGG pathways analysis of the downregulated circRNAs with parental transcripts. Only the top 20 enrichment KEGG pathways are shown in the figures.

To explore the biological processes that are potentially mediated by the identified DE circRNAs in the iCCA tissues, we performed the parental transcripts of these DE circRNAs to do the GO annotation analysis. The results showed that 736 GO terms were significantly enriched, such that the parental transcripts of the upregulated circRNAs were involved in the microtubule-based movement, cilium assembly, negative regulation of the organ growth and positive regulation of the protein kinase B signaling and apoptotic process in the Biological Process category and in the ATP-dependent microtubule motor activity, dynein intermediate chain binding, dynein light chain binding, dynein light intermediate chain binding and cadherin binding in the Molecular Function category (Figure 4A), while the parental transcripts of the downregulated circRNAs were involved in the generation of precursor metabolites and energy in the Biological Process category and the receptor binding, DNA binding transcription factor activity, zinc ion binding, identical protein binding and metal ion binding in the Molecular Function category (Figure 4B). A total of 112 KEGG pathways were associated with the parental transcripts of these DE circRNAs, such that the parental transcripts of the upregulated circRNAs were enriched in 44 KEGG pathways, including the “Huntington disease” (path:hsa05016) (Figure 4C), while the parental transcripts of the downregulated circRNAs were enriched in 76 KEGG pathways, including the “Complement and coagulation cascades” (path:hsa04610), “*Staphylococcus aureus* infection” (path:hsa05150), “Linoleic acid metabolism” (path:hsa00591), “Chemical carcinogenesis” (path:hsa05204) and “Arachidonic acid metabolism” (path:hsa00590) (Figure 4D).

### CircRNA-miRNA co-expression network

**FIGURE 5:**
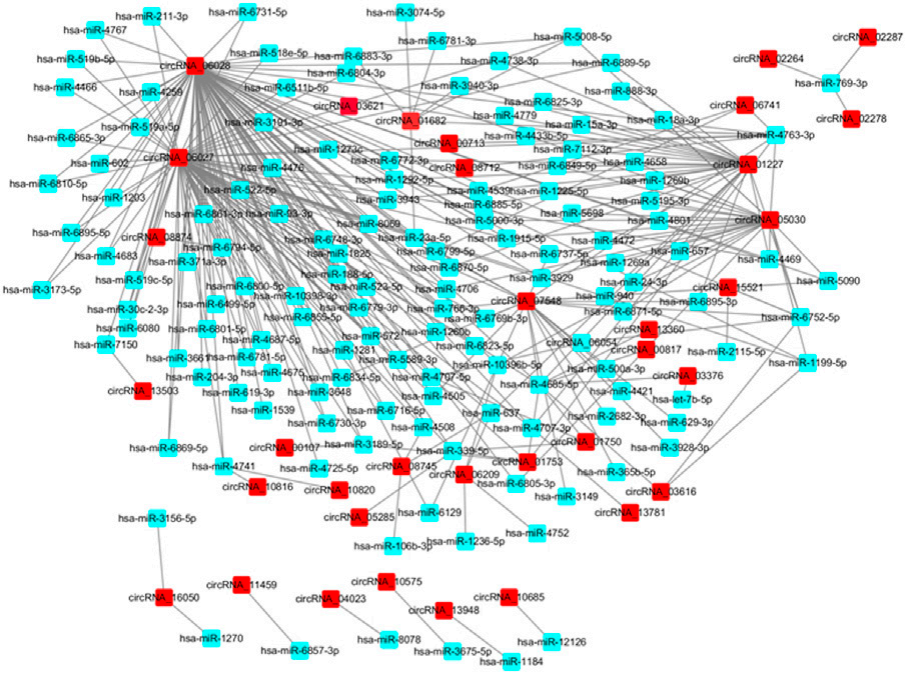
Network of the DE circRNA-DE miRNA co-expression.

One of the main biological roles of circRNA is the miRNA sponge. In this work, the target miRNAs of the identified DE circRNAs were predicted using the miRanda software. To construct the circRNA-miRNA co-expression network, the DE miRNAs were extracted from the iCCA and iCCAP tissues using small RNA sequencing data. According to the miRNA binding sites prediction data, the interaction between the DE circRNAs and miRNA was ranked by miRanda based on the *p*-value (Zhang et al., 2017). The interaction network of circRNA-miRNA showed that 300 DE miRNAs interacted with 60 DE circRNAs among the top 300 interaction pairs, which were predicted to be co-expressed in iCCA (Figure 5).

### Construction of ceRNA networks

**FIGURE 6:**
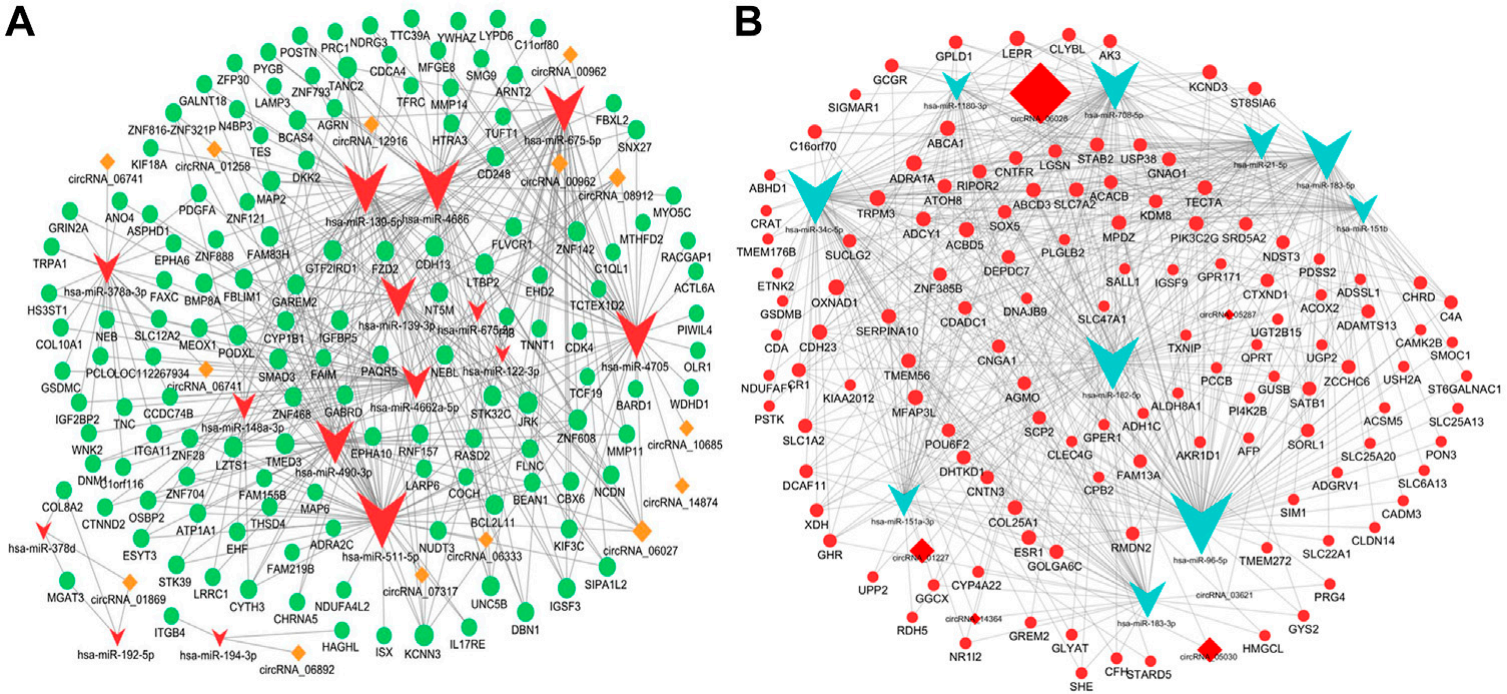
Construction of the ceRNA networks. **(A)** The upregulated circRNA-mediated ceRNA network. **(B)** The downregulated circRNA-mediated ceRNA network.

The ceRNA hypothesis was a novel approach to uncover the molecular pathogenesis of diseases. Aiming to further understand the potential functions of these circRNAs in the pathogenesis and progression of the iCCA tissues, we constructed the circRNA ceRNA networks using the DE mRNAs, miRNAs, and circRNAs. The DE miRNAs and mRNAs were extracted from the iCCA and iCCAP tissues using RNA sequencing data. According to the ceRNA hypothesis, the upregulated or downregulated mRNAs were chosen to construct the upregulated or downregulated circRNAs ceRNA networks, respectively. First, we constructed the ceRNA networks in the iCCA. According to the regulation of the target prediction, a total of 370 upregulated mRNAs, 399 downregulated miRNAs, and 29 upregulated circRNAs were found with an interaction correlation, and 563 downregulated mRNAs, 584 upregulated miRNAs and 21 downregulated circRNAs were found with an interaction correlation in the iCCA. According to the circRNA mediated ceRNA networks, a total of 121 downregulated mRNAs were identified in the downregulated circRNA ceRNA network (Figure 6A), and 137 upregulated mRNAs were identified in the upregulated circRNA ceRNA network (Figure 6B).

### Gene ontology and kyoto encyclopedia of genes and genomes analysis of the differential expressed circRNA-mediated ceRNAs in iCCA

**FIGURE 7:**
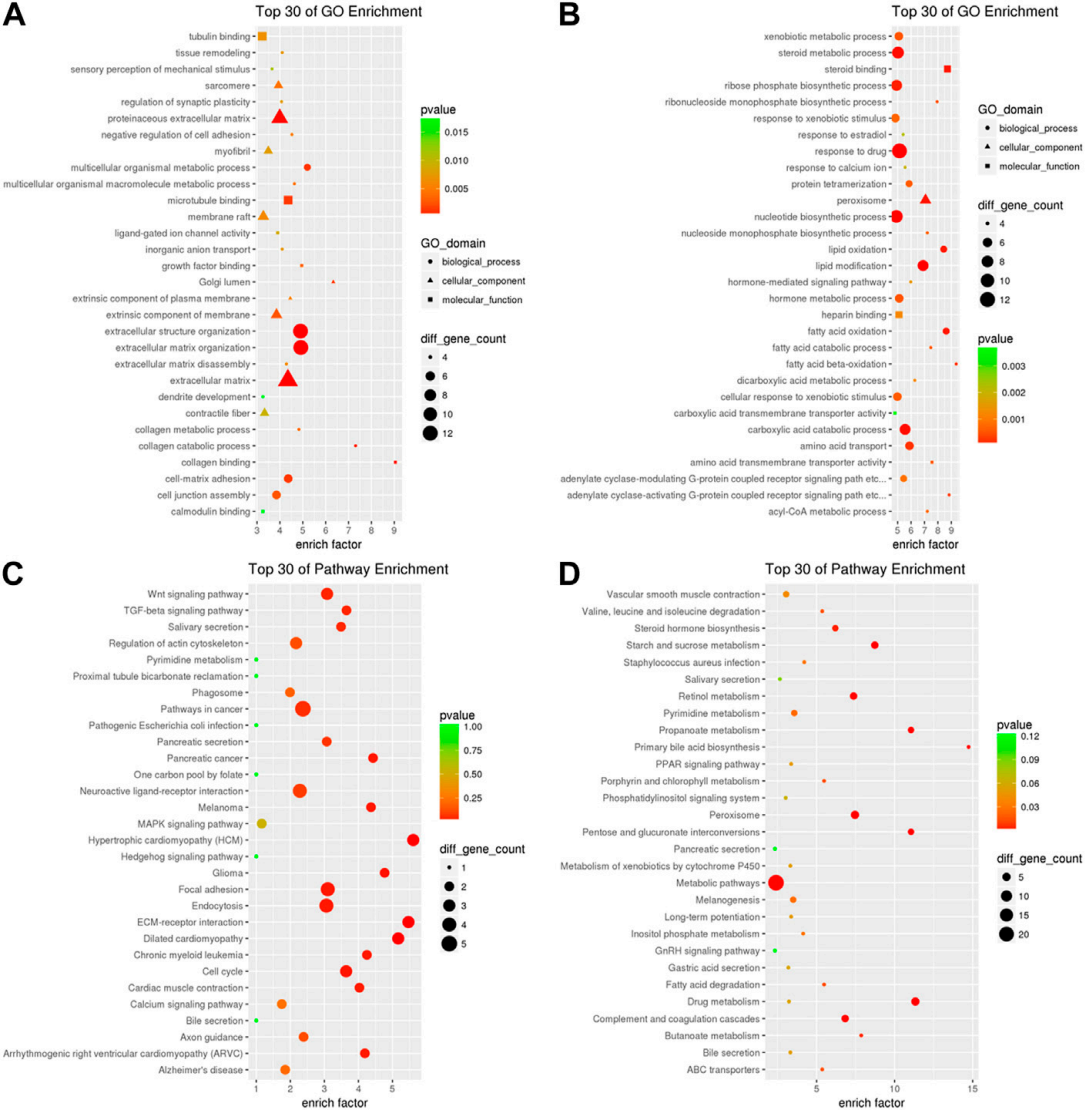
GO and KEGG analyses of the DE circRNA-mediated ceRNAs in iCCA. **(A)** The upregulated mRNAs in the upregulated circRNA-mediated ceRNAs were analyzed with GO analysis. **(B)** The downregulated mRNAs in the upregulated circRNA-mediated ceRNAs were analyzed with GO analysis. **(C)** The upregulated mRNAs in the upregulated circRNA-mediated ceRNAs were analyzed with KEGG analysis. **(D)** The downregulated mRNAs in the upregulated circRNA-mediated ceRNAs were analyzed with KEGG analysis.

In order to further uncover the potential roles of the DE circRNA-mediated ceRNAs in iCCA, we analyzed the upregulated mRNAs in the upregulated circRNA-mediated ceRNAs using GO analysis, and the results showed that the upregulated circRNA-mediated ceRNAs were related to the functions of microtubule binding, tubulin binding, ligand-gated ion channel activity, growth factor binding, collagen binding and calmodulin binding (*p* < 0.05, Figure 7A). On the other hand, the downregulated mRNA in the downregulated circRNA-mediated ceRNAs were involved in the functions of steroid binding, heparin binding, carboxylic acid transmembrane transporter activity and amino acid transmembrane transporter activity (*p* < 0.05, Figure 7B). Furthermore, to further understand the identified DE mRNAs in the circRNA-mediated ceRNAs that are related to the signaling pathways, the upregulated mRNAs in the upregulated circRNA-mediated ceRNAs were analyzed using the KEGG analysis, and the results showed that the upregulated circRNA-mediated ceRNAs were enriched in the pathways of cancer, “Wnt,” “TGF-beta,” “MAPK,” and “Endocytosis signaling pathway” (Figure 7C), and the downregulated mRNA in the downregulated circRNA-mediated ceRNAs were enriched in the metabolic pathways and the pathways of “Peroxisome,” “Drug metabolism,” “Complement and coagulation cascades,” and “Retinol metabolism” (Figure 7D). These results indicated that many important immunity and metabolic pathways that are related to mRNAs were mediated by the DE circRNAs *via* miRNAs in the progress or pathogenesis of the iCCA.

## Discussion

iCCA represents the second most common primary hepatic malignancy following HCC. It is reported that 10%–20% of the newly diagnosed liver cancer cases were diagnosed to be iCCA (Altaee et al., 1991). Since the clinical features, biochemical profiles and radiologic features are not specific in patients with iCCA, it is a great challenge for pathologists to diagnose iCCA in its early stage (Bledsoe et al., 2015; Omata et al., 2017). The surrounding interstitial connective tissue of the patients with iCCA could be easily infiltrated by iCCA, leading to a poor prognosis (Hamaoka et al., 2019). The molecular mechanisms underlying iCCA are still largely unknown. CircRNAs are a unique class of RNAs, which were reported to play crucial roles in the tumors’ oncogenesis and progression, or as biomarkers for the early diagnosis of cancers, including cholangiocarcinoma (Jiang et al., 2018; Xu et al., 2018; Xu et al., 2019a), HCC (Su et al., 2019; Zhu et al., 2019), oral squamous cell carcinoma (Zhao et al., 2019), gastric cancer (Lu et al., 2019) and non-small cell lung cancer (Wang et al., 2019c). An increasing number of studies suggest that circRNAs are novel RNA molecules with different biological functions and pathological implications (Xu et al., 2019b). The expression patterns of circRNAs were explored to elucidate the potential mechanisms of the circRNAs in the pathogenesis of iCCA through high-throughput sequencing. Using the Venn analysis, 117 DE circRNAs were identified from the iCCA compared with iCCAP. These DE circRNAs may be important regulatory molecules in the pathogenesis and progression of iCCA, and several ones were further verified by the real-time PCR and the reliability and validity of the sequencing data were confirmed in 10 iCCA and 10 iCCAP samples. The DE circRNAs may be used as novel non-invasive biomarkers for the early diagnosis of iCCA, which still needs large patient cohorts to be validated. GO and KEGG analyses were performed to investigate the potential roles of the DE circRNAs with their parental transcripts (Zhang et al., 2017; Qiu et al., 2019). In this study, the GO analysis revealed that the most significant enriched GO terms were the generation of precursor metabolites and energy and the positive regulation of the protein kinase B signaling and the apoptotic process in the Biological Process category, and the ATP-dependent microtubule motor activity, receptor binding, zinc ion binding, identical protein binding, metal ion binding and cadherin binding in the Molecular Function category. These enriched GO terms might be of great significance during the pathogenesis of iCCA. In the KEGG pathway analysis, several immune and cancer pathways were identified, which present a deep insight into the role of circRNAs in the pathogenesis of iCCA. The potential signaling pathways such as the “Huntington disease,” “Complement and coagulation cascades,” “*S. aureus* infection,” “Linoleic acid metabolism,” “Chemical carcinogenesis,” and “Arachidonic acid metabolism” were involved, which may play pivotal roles in the pathogenesis of iCCA and provide novel clues to explore the mechanism of circRNAs in the tumor oncogenesis and progression.

One of the most studied biological functions of the circRNAs is miRNA sponges, which were reported to modulate the post-transcriptional regulation in various cancer types, including HCC and iCCA. The targeted miRNAs of the DE circRNAs were predicted by a conserved seed-matching sequence. A total of 117 DE circRNAs were found to be combined with 1813 binding miRNAs, such that one circRNA had one or more binding sites to different miRNAs. The circRNA-miRNA interaction analysis might partly reveal the possible mechanisms of these circRNAs in the pathogenesis of iCCA. circRNA_01227 had 27 potential binding sites, including miR-4779, miR-6895-3p, miR-1292-5p, hsa-miR-4685-5p, hsa-miR-7112-3p, miR-6849-5p and many more. Among these involved miRNAs, many were related to cancer development and progression. miRNA-572 functions as an oncogene, and it is a potential biomarker for the diagnosis, treatment, and prognosis of renal cell carcinoma (Pan et al., 2018). The migration, invasion, and cell cycle of the glioma cells could be inhibited by hsa-let-7b-5p (Xi et al., 2019). miR-637 is a tumor suppressor in several cancer types, which plays a crucial role in melanoma progression (Li et al., 2018).

Moreover, two circRNA-miRNA-mRNA ceRNA networks were constructed with the sequencing data. The first network contained 399 downregulated miRNAs, 370 upregulated mRNAs, and 29 upregulated circRNAs, and the second one contained 21 downregulated circRNAs, 563 downregulated mRNAs, and 584 upregulated miRNAs. These data might help us to better insight into the regulatory of circRNAs-mediated ceRNA in iCCA. Therefore, the GO and KEGG analyses were performed on the upregulated and downregulated mRNAs mediated by the two ceRNA networks, and the results showed that many important immunity and metabolic pathways that are related to mRNAs were regulated by the DE circRNAs *via* miRNA in the progress or pathogenesis of the iCCA, especially the pathways of “Wnt,” “TGF-β,” and “MAPK signaling pathway.” The aberrant activation of Wnt/β-catenin signaling was closely related to the tumor malignancy and patient outcome in the majority of CCA cases. This pathway may represent a potential liver cancer treatment by targeting the signaling components (Wang et al., 2019b). The TGF-β signaling pathway influences diverse physiological processes. When the activity of this pathway is excessive and uncontrolled, pathological conditions, such as fibrotic diseases and tumorigenesis, may arise (Papoutsoglou et al., 2019). The TGF-β pathway also exhibits immune-suppressive effects, which might help the cancer cells to escape the immune surveillance (Papoutsoglou et al., 2019). The activation of P38/MAPK signaling is associated with inflammation and tumor malignant progression (Cecil et al., 2005; Sparvero et al., 2009; Maletzki et al., 2012). These results suggested that some circRNAs that are related to the pathways of “Wnt,” “TGF-β,” and “MAPK signaling pathway” will be developed to act as effective therapeutic targets for iCCA.

In summary, the circRNA expression profiles were investigated using high-throughput sequencing to explore their potential regulatory mechanisms in iCCA. 117 DE circRNAs were identified by comparing iCCA with iCCAP. Based on the parental transcripts of these DE circRNAs, the GO and KEGG analyses were applied to predict the most significant enriched GO terms and their potential regulatory relationships. Meanwhile, the potential ceRNA networks were applied to annotate the potential functions of these DE circRNAs. Furthermore, to uncover the potential roles of the DE circRNA-mediated ceRNAs in iCCA, the upregulated and downregulated mRNAs in the upregulated and downregulated circRNA-mediated ceRNAs were analyzed using GO and KEGG analyses. Some circRNAs that are related to the pathways of “Wnt,” “TGF-β,” and “MAPK signaling pathway” will be developed to act as an effective therapeutic target for iCCA in the future. These data will provide novel insight into the regulatory mechanism of the circRNAs in tumor oncogenesis and the progression of iCCA. However, the molecular mechanism of these circRNAs in the epidemiology and carcinogenesis of iCCA still needs further investigation.

## Data Availability

The datasets presented in this study can be found in online repositories. The names of the repository/repositories and accession number(s) can be found below: NCBI Bioproject accession numbers: PRJNA763019 and PRJNA763017.

## Abbreviations

ICCA, intrahepatic cholangiocarcinoma; pCCA, perihilar cholangiocarcinoma; dCCA, distal cholangiocarcinoma; HCC, hepatocellular carcinoma; circRNAs, Circular RNAs; iCCAP, adjacent normal tissues; ceRNA, Competitive endogenous RNA; DE, differentially expressed.
